# Iodine Fortification of a Fermented Dairy Product: Development and Quality Assessment

**DOI:** 10.3390/foods15122096

**Published:** 2026-06-10

**Authors:** Aigul Tayeva, Talgat Kulazhanov, Fatima Dikhanbayeva, Zhadyra Imangaliyeva, Rimma Elemanova, Mamura Absalimova, Aliya Kengesova, Dariya Tapalova, Ulzhan Anarbekova

**Affiliations:** 1 Department of Food Technology, Almaty Technological University, 100 Tole bi Str., Almaty 050012, Kazakhstan; aigul.taeva@gmail.com (A.T.); tkulazhanov_atu@mail.ru (T.K.); fatima6363@mail.ru (F.D.); kengesova93@mail.ru (A.K.); dariyaxt@gmail.com (D.T.); anarbekova1603@mail.ru (U.A.); 2 Food Science and Technology, Kyrgyz State Technical University named after I. Razzakov, 66 Ch. Aitmatov Ave., Bishkek 720044, Kyrgyzstan; elemanova@kstu.kg

**Keywords:** fermented dairy product, iodine, potassium iodide, iodocasein, fortification

## Abstract

Iodine deficiency remains a major nutritional concern worldwide, and fermented dairy products are considered promising vehicles for iodine fortification. However, the chemical form of iodine may influence the physicochemical and microbiological stability of fermented dairy systems. This study aimed to compare potassium iodide and iodocasein as iodine sources for the fortification of a fermented dairy product and to evaluate their effects on product quality and iodine retention during refrigerated storage. Three formulations were produced: a control sample without iodine fortification and samples fortified with potassium iodide or iodocasein at a level of 25 µg iodine per 100 g of product. Samples were stored at 4 ± 1 °C for 7 days. Changes in pH, titratable acidity, syneresis, viscosity, viable counts of starter lactic acid bacteria, iodine retention, and sensory properties were evaluated during storage. The results showed that the iodine source significantly affected product stability. The potassium iodide-fortified sample exhibited greater post-acidification, increased syneresis, lower viscosity, and a more pronounced reduction in viable lactic acid bacteria during storage. In contrast, the iodocasein-fortified sample maintained physicochemical and microbiological characteristics closer to the control and demonstrated higher iodine retention. Fortification at the studied level did not significantly affect the basic composition or amino acid profile of the product. The findings indicate that iodocasein can improve iodine stability while preserving the quality characteristics of fermented dairy products, supporting its potential application in the development of iodine-enriched functional dairy foods.

## 1. Introduction

Adequate iodine intake remains important for maintaining normal thyroid activity and metabolic regulation. According to Hatch-McChesney and Lieberman [1], insufficient iodine consumption is associated with impaired thyroid function, reduced cognitive performance, and unfavorable developmental outcomes, particularly in vulnerable population groups. Wu et al. [2] also reported that iodine deficiency remains a relevant nutritional problem in many regions despite existing preventive measures. Gunnarsdóttir and Brantsæter [3] noted that current changes in dietary habits may contribute to unstable iodine intake, thereby increasing interest in alternative dietary iodine sources.

In accordance with the guidelines and principles of FAO/WHO, nutritionists recommend consuming 2–3 portions of dairy products per day. One portion is defined as approximately 1 glass (200–250 mL) of kefir or yoghurt (sugar-free). According to the nationally approved, scientifically based physiological dietary intake standards in Kazakhstan, there are recommended dietary intake levels for certain age and gender groups of the male population (children, adolescents, the elderly), in kilograms per year. For adults, the minimum intake of fermented milk products is 39.3–46 kg per year, with a daily requirement of 200–500 mL. The recommended daily intake of iodine is 127 µg; according to the FAO/WHO, it is 75 µg.

The physiological effect of iodine depends not only on its concentration in fortified foods but also on its release and availability during digestion. After ingestion, iodine compounds are mainly converted into iodide, absorbed in the upper gastrointestinal tract, and utilized by the thyroid gland for the synthesis of thyroxine (T4) and triiodothyronine (T3), whereas excess iodine is primarily eliminated via urine [4,5]. Consequently, evaluation of iodine-fortified fermented dairy products should also consider the bioaccessibility of iodine from different chemical forms, since variations in iodine availability may affect thyroid hormone balance and metabolic processes [6].

Salt iodization remains one of the most widely applied approaches for the prevention of iodine deficiency disorders [7]. However, as discussed by Rigutto-Farebrother and Zimmermann [8], modern nutritional policies increasingly emphasize sodium reduction, creating interest in alternative food-based iodine delivery systems. Therefore, the development of fortified staple foods capable of providing adequate iodine intake without increasing sodium consumption is considered a promising direction in preventive nutrition [9].

Dairy products contribute substantially to iodine intake in many populations [10]. Nevertheless, iodine concentration in milk and dairy foods may vary depending on feeding strategies, seasonal factors, hygienic practices, and technological processing conditions [11]. Machado et al. [12] reported that the iodine value of dairy products should be evaluated with consideration of processing conditions and iodine retention after production rather than solely based on raw milk composition. Similar observations were presented by Sakai et al. [13], who demonstrated variability in iodine levels even within identical product categories. According to Khandakar and Islam [14], the final iodine concentration in milk is influenced by multiple biological and technological factors, which should be considered during the development of iodine-enriched dairy products.

Fermented dairy products are regarded as promising carriers for micronutrient fortification because of their nutritional value, digestibility, and high consumer acceptance [15,16]. At the same time, fermented milk systems represent structurally complex protein gels whose properties are affected by formulation composition, thermal treatment, fermentation conditions, and storage duration. Product quality in such systems is closely associated with their ability to retain moisture and maintain an integrated gel network throughout storage. Arab et al. [17] identified syneresis as one of the principal indicators of product instability due to its relationship with water-holding capacity. Jørgensen et al. [18] further demonstrated that both milk composition and processing conditions influence gel structure and texture formation. Atik et al. [19] also reported that rheological behavior in fermented milk systems is strongly dependent on formulation characteristics and storage-related changes. These observations suggest that relatively small formulation changes can influence both structural and sensory attributes of fermented milk products.

The high sensitivity of fermented milk matrices to formulation changes is especially relevant for micronutrient fortification. Waweru et al. [20] reported that modifications in fermented milk composition may influence physicochemical, microbiological, and sensory characteristics during refrigerated storage. Ilić et al. [21] observed that variations in starter culture combinations affected rheological behavior and sensory properties of fermented products. Shi et al. [22] demonstrated that compositional modifications can alter acidity, aroma profile, texture, and water-holding capacity. According to Ahmad et al. [23], incorporation of additional ingredients into dairy matrices may significantly affect viscosity, texture, and consumer acceptability. Nole-Jaramillo et al. [24] confirmed that mineral fortification can lead to measurable changes in compositional and sensory characteristics even when the desired enrichment level is achieved.

In this regard, the chemical form of iodine may play an important role in the preservation of product properties in fortified fermented dairy products. Potassium iodide is commonly used as an inorganic iodine source because of its availability and ease of application. However, in acidified protein systems, inorganic iodine compounds may influence ionic interactions, post-fermentation processes, and the integrity of the protein network during storage. By contrast, iodocasein represents a protein-bound iodine form that may demonstrate greater compatibility with the dairy matrix and improved retention behavior in fermented systems. Nevertheless, comparative information regarding the behavior of different iodine forms in fermented dairy products remains limited.

At the same time, the use of iodocasein requires a cautious interpretation from a nutritional and safety perspective. Since iodocasein is based on casein, it is associated with a milk-protein carrier; casein fractions are recognized among cow’s milk allergens [25,26]. Although the fermented dairy matrix itself naturally contains milk proteins, this point should be acknowledged when discussing product applicability, labelling, and potential limitations for consumers with cow’s milk protein allergy. For a protein-bound iodine source, the release of iodine during digestion may depend on enzymatic hydrolysis of the casein carrier, which can make its biological response less directly predictable than that of simple inorganic iodide salts.

Thus, fermented dairy products can be considered promising matrices for iodine fortification, while the comparative evaluation of different iodine forms in such systems remains insufficiently explored. From a scientific perspective, it is important to compare potassium iodide as a conventional inorganic iodine source with iodocasein as a protein-bound form of the micronutrient in terms of their effects on physicochemical properties, structural-mechanical behavior, microbiological parameters, and organoleptic characteristics of the product. From a practical standpoint, such a comparison may facilitate the selection of an iodine form that ensures not only the target level of fortification but also acceptable storage performance and product quality throughout refrigerated storage.

In this regard, the aim of the present study was to compare potassium iodide and iodocasein as sources of iodine for the fortification of a fermented dairy product, as well as to evaluate their effects on product quality and storage behavior. The working hypothesis of the study was that the chemical form of iodine would determine not only the retention of the micronutrient in the final product but also the direction and magnitude of changes in acidity, structure, microflora, and sensory properties during refrigerated storage.

## 2. Materials and Methods

### 2.1. Materials

Cow’s milk suitable for the production of fermented milk products was used as the main raw material. The cow’s milk was sourced directly from the ‘Amantai’ farm in the village of Uzynagash, Zhambyl District, Almaty Region, Kazakhstan, and was obtained immediately after milking (raw bulk milk) and transported under refrigerated conditions to the laboratory.

Milk collection was carried out during the spring–summer period under pasture-based feeding conditions, when cows were predominantly fed fresh forage with partial supplementation of standard farm rations. It should be noted that seasonal feeding and housing conditions may influence milk composition; however, all experimental batches were obtained from the same farm under the same production conditions to ensure uniformity of raw material.

A starter culture Danisco YO-MIX^®^ 495 (Danisco, Dangé-Saint-Romain, France) was used for the direct fermentation of the fermented milk product in a vat. The starter culture consists of cultures (*Streptococcus thermophilus* + *Lactobacillus delbrueckii* subsp. *bulgaricus*), manufactured in France. Two chemically distinct forms of iodine were used as sources of iodine: potassium iodide, manufactured by Vitamir, Moscow, Russia, as the inorganic form; and the complex food supplement ‘Iodcasein’, manufactured in Russia by the company «Evalar», Biysk, Altai Krai, Russia, as the protein-bound form of this trace element.

Prior to fermentation, the raw milk was subjected to basic quality control analyses, including determination of pH, titratable acidity, fat content, protein content, and dry matter, to ensure compliance with technological requirements for fermented dairy production. The milk used in all experimental variants demonstrated uniform physicochemical characteristics across batches.

Three samples of a fermented milk product were prepared: a control sample without any iodine-containing ingredients (Control), a sample enriched with potassium iodide (KI), and a sample enriched with iodocasein (I-Cas). For each variant, three independent production batches were prepared under identical laboratory conditions. The finished samples were stored at a temperature of 4 ± 1 °C for 7 days. Samples for analysis were taken on the 1st, 3rd, 5th and 7th days of storage.

### 2.2. Fermented Dairy Product Preparation

Prior to processing, the milk was filtered and standardized. The milk was subjected to heat treatment at 90 ± 2 °C for 5 min, followed by cooling to the inoculation temperature of 40–42 °C. The prepared milk base was then divided into three portions. No fortificants were added to the control sample.

For the KI variant, a pre-prepared potassium iodide solution was added to the pasteurised milk base under continuous stirring. For the iodocasein sample (I-Cas), the preparation was preliminarily dispersed in a portion of the pasteurised milk base to obtain a homogeneous suspension and subsequently incorporated into the total volume of the mixture. The dosage of iodine-containing fortificants was determined based on the target iodine content in the final product (25 μg/100 g), the batch mass, and the iodine content of the fortificant used. The iodocasein preparation contained 10% iodine. Therefore, to achieve a fortification level of 25 μg iodine/100 g, 250 μg of iodocasein preparation was added per 100 g of product. In WHO/FAO documents, potassium iodide is considered one of the standard iodine-containing fortificants. The practical conditions for the use of iodocasein were established in accordance with methodological guidelines for its application in milk and dairy products.

After the addition of fortificants, the starter culture was introduced into all variants. Fermentation was carried out at 40–42 °C until an active acidity of pH 4.6 ± 0.05 was reached. Upon completion of fermentation, the product was packaged into sterile polymer cups (100 g), cooled to 4 °C for 1 h, and stored under refrigerated conditions for further analysis.

### 2.3. Determination of Acidity

The active acidity of fermented dairy product samples was determined by a potentiometric method using a digital pH meter (HM Digital PH-200, HM Digital, Inc., Culver City, CA, USA), following an approach similar to that described by Marlapati et al. [27]. Prior to measurements, the instrument was calibrated using standard buffer solutions (Hanna Instruments, Woonsocket, RI, USA) at pH 4.00 and pH 7.00. Measurements were carried out at 20 ± 2 °C in triplicate, and the results were expressed in pH units.

Titratable acidity was determined by a potentiometric method in accordance with ISO/TS 11869:2012 (IDF/RM 150:2012) [28]. The method is based on the neutralization of acids present in the sample with 0.1 mol/L NaOH (Merck KGaA, Darmstadt, Germany) to an endpoint of pH 8.30, with potentiometric detection of the equivalence point. The results were expressed in degrees Turner (°T).

### 2.4. Rheological Properties

Syneresis analysis of the different fermented dairy product samples stored at 4 ± 1 °C was performed on days 1, 3, 5, and 7 in quadruplicate by centrifugation according to the methodology proposed by Zbikowska et al. [29] with modifications by Saleh et al. [30]. For this purpose, 5.0 g of the samples was weighed and centrifuged at 2500 rpm for 20 min at 7 ± 1 °C in the refrigerated centrifuge (OPn-3M, Dastan JSC, Bishkek, Kyrgyzstan). The supernatant was collected and weighed, and the syneresis index was determined according to Equation (1):

(1) $$Syneresis(\%)=\frac{S}{W}\times 100\%\;$$

where *S* is mass of supernatant (g), *W* is total sample weight (g).

Apparent viscosity was measured using a rotational viscometer with a touchscreen display (RV-2TDV, Lamy Rheology Instruments, Champagne-au-Mont-d’Or, France) at 7 ± 1 °C and a spindle speed of 50 rpm. The temperature was maintained using a thermostatically controlled water bath. Readings were recorded after 30 s for each sample. Appropriate spindles were selected to maintain the torque within the range of 25–75%. All measurements were performed in triplicate [31,32].

### 2.5. Physicochemical Properties

Mass fraction of protein was determined by the Kjeldahl method according to ISO 8968-1:2014 [33]. The method is based on the determination of total nitrogen followed by conversion to protein. Fat content was determined by the gravimetric method according to ISO 1211:2010 [34]. Ash content was determined by dry ashing to constant weight in a muffle furnace (LOIP LF-7/13-G1, LOIP Ltd., Saint Petersburg, Russia) according to ISO 936:1998 [35]. Carbohydrate content on a dry matter basis was calculated by difference as 100 minus the sum of protein, fat, and ash contents, expressed as g/100 g of product.

Sampling for physicochemical analysis was carried out on day 1 of storage. All measurements were performed in triplicate, followed by calculation of the mean value and standard deviation.

### 2.6. Microbiological Analysis

Sample preparation, preparation of the initial suspension, and serial tenfold dilutions were performed in accordance with ISO 6887-5 [36]. General requirements for microbiological testing were followed in accordance with ISO 7218 [37]. The counts of characteristic microflora of the fermented dairy product were determined by the colony count method according to ISO 7889:2003/IDF 117:2003 [38], intended for the enumeration of *Lactobacillus delbrueckii* subsp. *bulgaricus* and *Streptococcus thermophilus*.

For analysis, 10 g of the product were aseptically sampled, an initial suspension was prepared, serial tenfold dilutions were performed, and aliquots were plated onto appropriate culture media. After incubation, the grown colonies were counted, and the results were expressed as logarithms of colony-forming units per gram of product (log CFUs/g).

### 2.7. Iodine Detection and Quantification

Iodine content in fermented dairy product samples was determined by inductively coupled plasma mass spectrometry (ICP-MS) using an Agilent 7900 ICP-MS spectrometer (Agilent Technologies, Tokyo, Japan) according to the EN 15111:2007 standard [39]. Samples were homogenized, and 100–500 mg (dry weight) was weighed into airtight vessels. For alkaline extraction, 5 mL of deionized water and 1 mL of 25% tetramethylammonium hydroxide (TMAH) (Merck KGaA, Darmstadt, Germany) were added. The mixture was incubated at (90 ± 3) °C for 3 h. After cooling, the extract was diluted to 25 mL and filtered or centrifuged. An aliquot of the extract was mixed with a tellurium internal standard (Merck KGaA, Darmstadt, Germany) and diluted prior to analysis. Calibration solutions (5–50 µg/L) were prepared under the same conditions. ICP-MS measurements were performed using argon plasma, monitoring iodine at *m*/*z* 127 with internal standard correction. Results were calculated considering dilution factors and sample mass.

### 2.8. Sensory Evaluation

Sensory evaluation was conducted using hedonic rating and hedonic quality tests, involving 20 faculty members from the Department of Food Technology, in accordance with the methodology described in [40], with minor modifications. Sensory analysis was performed on days 1 and 7 of storage. The hedonic rating test included the evaluation of color, taste, aroma, texture, and overall acceptability, whereas the hedonic quality test assessed aftertaste, homogeneity, iodine flavor, sweetness, dairy aroma, and consistency.

All evaluations were carried out individually by each panelist using a 5-point scale, with results recorded in standardized score sheets.

The study complied with the ethical principles for research involving human participants outlined in the Declaration of Helsinki [41].

### 2.9. Amino Acid Content

The qualitative and quantitative amino acid composition of the fermented dairy product samples was determined by high-performance liquid chromatography (HPLC) using an Agilent 1260 Infinity II HPLC system (Agilent Technologies, Santa Clara, CA, USA) equipped with a diode-array detector (DAD). Separation was performed on a ZORBAX Eclipse AAA column (4.6 × 150 mm, 5 μm particle size, Agilent Technologies, USA) maintained at 40 °C. For the determination of total amino acids, an accurately weighed portion of the lyophilized sample extract (~100 mg) was dissolved in 5 mL of 40% ethanol and incubated in an ultrasonic bath for 10 min. Aliquots of 0.1–0.2 mL was transferred into test tubes and evaporated to dryness in a water bath at 60 °C. Then, 0.10 mL of 0.15 mol/L sodium hydroxide (Merck KGaA, Darmstadt, Germany) was added to the dried residue and mixed thoroughly. After that, 0.35 mL of phenylisothiocyanate solution (Merck KGaA, Darmstadt, Germany) in isopropyl alcohol and 0.05 mL of bidistilled water were added. The reaction mixture was mixed thoroughly, left at room temperature for 20 min, and then evaporated to dryness at 65 °C. The dry residue was dissolved in 1 mL of bidistilled water, and the resulting solution was subjected to chromatographic analysis [42]. The mobile phase consisted of solvent A (0.1 M sodium acetate buffer, pH 6.5 (Merck KGaA, Darmstadt, Germany)) and solvent B (acetonitrile: methanol, 45:45:10, *v*/*v*/*v* (Merck KGaA, Darmstadt, Germany)). Amino acid separation was carried out using gradient elution at a flow rate of 1.0 mL/min. Detection was performed at 254 nm.

### 2.10. Statistical Analysis

Statistical analysis of the experimental data was conducted using Statistica 13.0 (TIBCO Software Inc., Palo Alto, CA, USA) for Windows. The effects of sample type and storage duration on pH, titratable acidity, syneresis, viscosity, lactic acid bacteria count, and iodine content were assessed by two-way analysis of variance (ANOVA), followed by Tukey’s honestly significant difference (HSD) post hoc test. Differences in physicochemical composition determined on Day 1 of storage, were evaluated using one-way ANOVA followed by Tukey’s HSD test. Sensory evaluation data obtained on Days 1 and 7 were analyzed separately for each storage day using one-way ANOVA. Differences were considered statistically significant at *p* < 0.05.

## 3. Results and Discussion

### 3.1. pH

During the study, all samples exhibited a gradual decrease in pH accompanied by a simultaneous increase in titratable acidity (Table 1), indicating the progression of post-acidification typical of fermented dairy products [43]. The most pronounced changes in both parameters were observed in the KI sample, whereas the I-Cas variant-maintained values closer to those of the control sample.

In the control sample, pH decreased from 4.64 ± 0.02 on day 1 to 4.26 ± 0.03 on day 7, while titratable acidity increased from 78.4 ± 1.4 to 100.3 ± 1.9 °T. In the KI variant, the changes in both parameters were the most pronounced: pH decreased from 4.63 ± 0.02 to 4.17 ± 0.02, whereas titratable acidity increased from 81.2 ± 1.5 to 108.2 ± 2.0 °T. The I-Cas sample occupied an intermediate position and was characterized by a more moderate change in acidity compared to KI and by values closer to those of the control.

Two-way analysis of variance for pH revealed a statistically significant effect of storage time (*p* < 0.05), whereas the main effect of sample type was not significant (*p* > 0.05). However, pairwise comparisons demonstrated significant differences between samples at specific storage days, particularly on days 5 and 7, when the KI sample exhibited significantly lower pH values compared to the Control and I-Cas samples. These results indicate that the overall decrease in pH was primarily associated with storage duration, while differences between formulations became evident only at later stages of storage.

The obtained results are consistent with those reported by Tomovska et al. [44], who demonstrated that changes in formulation and storage conditions can significantly affect pH and titratable acidity in fermented dairy systems, and further showed that modifications in the composition of the dairy matrix are reflected in acidity parameters and the state of the protein structure [45]. Similarly, Huppertz et al. [46] reported that mineral fortification may be accompanied by changes in the physicochemical and sensory characteristics of the product, including acidity parameters.

The more pronounced decrease in pH and the most intensive increase in titratable acidity observed in the KI variant may indicate a stronger influence of the inorganic form of iodine on the ionic equilibrium of the dairy system and the progression of residual enzymatic processes [47]. In contrast, the more moderate changes observed in the I-Cas sample suggest that the protein-bound form of iodine interfered to a lesser extent with post-fermentation processes and was more compatible with the dairy matrix [48].

It should also be noted that an excessive increase in acidity can contribute to the destabilisation of the protein gel network, leading to increased syneresis and a deterioration in the product’s texture [49]. In the present study, the lower rate of subsequent acidity increase observed in the I-Cas sample corresponded to its more stable organoleptic and structural characteristics during storage, indicating improved compatibility of the protein-bound form of iodine with the matrix of the fermented milk product.

Stabilising acidity during storage is of practical importance, as an increase in acidity is one of the main factors limiting the shelf life and consumer acceptability of fermented milk products [50]. The results obtained indicate that iodocasein can provide a more controlled introduction of iodine into fermented milk systems without causing significant shifts in acidity parameters. This may contribute to improved storage stability and the preservation of organoleptic qualities during refrigerated storage.

Thus, the results for active and titratable acidity confirm that the chemical form of iodine is an important factor determining the nature of post-fermentation changes in fermented dairy products. Under the conditions studied, iodocasein provided a more stable acidity profile compared to potassium iodide, suggesting that the protein-bound form of iodine may represent a more technologically compatible fortification approach for fermented dairy systems.

### 3.2. Rheological Properties

**Figure 1:**
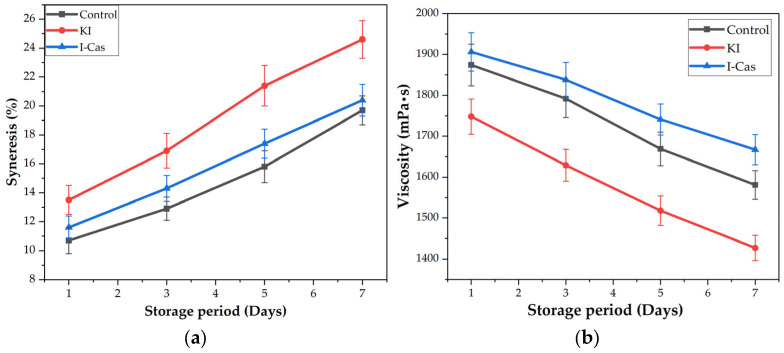
Changes in syneresis (**a**) and viscosity (**b**) of control and experimental samples of the fermented dairy product during storage. Control fermented dairy product without iodine fortification; KI, sample fortified with potassium iodide; I-Cas, sample fortified with iodocasein.

During refrigerated storage, all samples exhibited increased syneresis and decreased viscosity (Figure 1). The KI sample consistently showed the highest whey separation, whereas the I-Cas variant remained closer to the control throughout storage (Figure 1a).

Syneresis increased in all samples over time. In the control, it rose from 10.7 ± 0.9% on day 1 to 19.7 ± 1.0% on day 7. In the KI sample, values increased from 13.5 ± 1.0% to 24.6 ± 1.3%, while the I-Cas variant showed a smaller increase, from 11.6 ± 0.8% to 20.4 ± 1.1%.

The higher syneresis observed in the KI sample indicates weaker water retention within the gel structure during storage. In contrast, the lower whey separation in the I-Cas sample suggests that protein-bound iodine interacted more favorably with the dairy matrix, contributing to better preservation of the gel network.

These findings agree with previous studies on fermented dairy products. Akshit et al. reported that syneresis is strongly influenced by formulation composition and the condition of the protein network during storage [51]. Carrillo-López et al. further noted that increased whey separation reflects reduced water-holding capacity and progressive weakening of the gel structure [52].

Therefore, under the conditions of this study, iodocasein demonstrated a more stable whey separation profile than potassium iodide, indicating better suitability for iodine fortification of fermented dairy products.

The KI sample also showed the lowest viscosity values during storage, whereas the I-Cas variant maintained higher viscosity throughout the observation period.

In the control sample, viscosity decreased from 1874 ± 51.0 mPa·s on day 1 to 1581 ± 35.0 mPa·s on day 7, corresponding to a reduction of approximately 15.6%. A greater decline was observed in the KI sample, where viscosity decreased from 1748 ± 43.0 to 1427 ± 31.0 mPa·s (18.4%). Since potassium iodide is a low-molecular-weight compound, it likely does not contribute to reinforcement of the protein network, which may explain the more pronounced decrease in viscosity.

By contrast, the I-Cas variant maintained higher viscosity values throughout storage, decreasing from 1906 ± 47.0 to 1667 ± 37.0 mPa·s, corresponding to a smaller reduction of approximately 12.5%. This effect may be associated with the incorporation of iodine into the casein structure, which could promote stronger protein–protein interactions within the gel matrix.

The viscosity data are consistent with previous reports on fermented dairy systems. Hamed et al. [53] showed that higher viscosity is generally associated with lower syneresis and improved texture stability. Hoxha et al. [54] also described an inverse relationship between whey separation and viscosity in fermented milk gels. In addition, Robles-García et al. [55] demonstrated that reinforcement of the protein matrix contributes to reduced syneresis and improved preservation of product structure during storage.

Control of syneresis and viscosity is important for fermented dairy products because these parameters strongly affect texture, appearance, and shelf-life stability [56]. Excessive whey separation is commonly regarded as a quality defect that negatively influences consumer acceptance. In this regard, the improved rheological stability observed in the I-Cas sample indicates that protein-bound iodine may be advantageous for the production of iodine-fortified fermented dairy products with preserved textural characteristics during storage.

Overall, the results demonstrate that the chemical form of iodine significantly affected the rheological behavior of the fermented dairy product. Compared with potassium iodide, iodocasein provided lower syneresis and better viscosity retention throughout storage. The absence of a casein-only control group should be considered a limitation of the present study. Therefore, the improved rheological stability observed in the I-Cas sample cannot be attributed exclusively to the iodine moiety itself and may partly reflect matrix-related and structural effects associated with the casein carrier.

### 3.3. Physicochemical Properties

**Figure 2:**
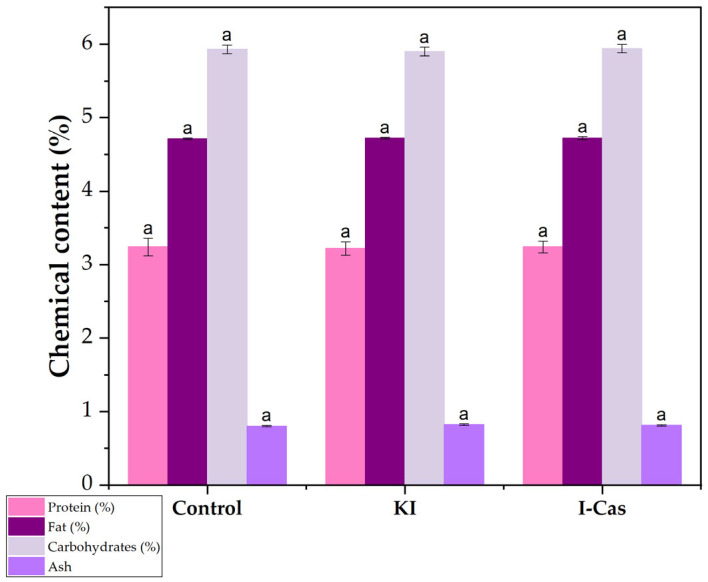
Physicochemical properties of the experimental samples on Day 1 of storage. Control fermented dairy product without iodine fortification; KI, sample fortified with potassium iodide; I-Cas, sample fortified with iodocasein. The same letter (‘a’) above bars indicates no statistically significant differences were observed among the samples (*p* > 0.05).

Preliminary evaluation of the chemical composition showed that the addition of iodine-containing components at the applied dosages did not result in pronounced changes in the basic nutrient profile of the product (Figure 2). In all studied variants, the contents of protein, fat, carbohydrates, and ash remained comparable, and the observed variations were within the limits of experimental variability. This suggests that at a dosage of 25 μg of iodine per 100 g of product, both forms of the fortificant did not have a significant effect on the proximate composition of the fermented dairy product.

The obtained data are in good agreement with the literature on functional fortification of fermented dairy products. Saleem et al., in a review study, note that the addition of any supplementary ingredients may modify the physicochemical, rheological, microbiological, and sensory properties of fermented dairy products; however, the extent of these changes depends on the nature of the additive and its dosage [57]. At low levels of mineral fortification, the basic composition of the product often remains stable. Thus, Zommara et al. [58] demonstrated that fortification of fermented dairy products with chromium picolinate at low doses did not result in statistically significant changes in physicochemical, rheological, microbiological, or sensory properties compared to the control. Similarly, Turek et al. [59] reported that the addition of microencapsulated bioactive lipid components did not lead to a substantial deterioration of physicochemical and sensory characteristics of fermented dairy products.

The preservation of the core nutrient profile following iodine fortification is a key advantage for the development of functional fermented dairy products. The results obtained show that iodine fortification at the dosage used allows the micronutrient value of the product to be increased without significantly altering its traditional nutritional profile, including the content of proteins, fats and carbohydrates. This is particularly relevant for preventive dietary strategies aimed at correcting iodine deficiency whilst maintaining the familiar characteristics of the diet and the consumer acceptability of fermented milk products.

Furthermore, the absence of significant physicochemical changes suggests that the addition of iodine via these fortifiers at low doses could be incorporated into standard dairy production processes without the need for major technological modifications. Consequently, the formulations studied can be considered promising carriers for delivering iodine to the diet within the context of functional dairy systems.

### 3.4. Changes in Lactic Acid Bacteria Counts During Storage

During refrigerated storage, all samples showed a gradual decrease in lactic acid bacteria counts, which is characteristic of post-fermentation changes in fermented dairy products. However, the magnitude of this decline depended on the iodine form added. The lowest counts throughout storage were observed in the KI sample, whereas the I-Cas sample remained closer to the control.

In the control sample, lactic acid bacteria counts decreased from 8.23 ± 0.07 to 7.70 ± 0.07 log CFU/g between days 1 and 7, corresponding to a 0.53 log reduction (6.4%). A more pronounced decline was observed in the KI sample, where counts decreased from 8.11 ± 0.08 to 7.44 ± 0.08 log CFU/g, corresponding to a 0.67 log reduction (8.3%). In contrast, the I-Cas sample demonstrated greater microbial stability, with counts decreasing from 8.26 ± 0.06 to 7.84 ± 0.06 log CFU/g, corresponding to a 0.42 log reduction (5.1%). Differences between the KI sample and the other variants became apparent from day 3 and were most pronounced by day 7.

These results are consistent with previous studies reporting a progressive decline in yogurt starter cultures during refrigerated storage, the extent of which depends on product composition and added functional ingredients [60,61,62]. The greater reduction observed in the KI sample may indicate less favorable conditions for maintaining starter culture viability in the presence of inorganic iodine. This interpretation is supported by the higher post-acidification, increased syneresis, and lower viscosity observed in the KI variant during storage. In contrast, the higher retention of lactic acid bacteria in the I-Cas sample suggests that the protein-bound form of iodine exerted a milder effect on the fermented dairy matrix, contributing to improved microbiological stability.

**Figure 3:**
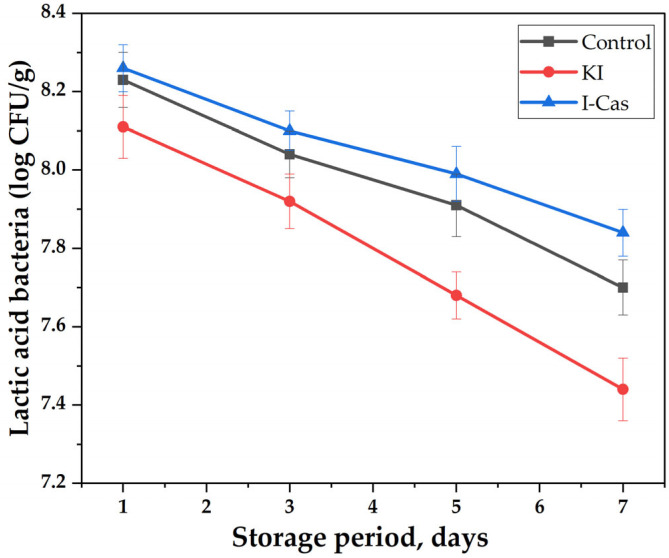
Changes in the counts of characteristic starter microflora in control and fortified fermented dairy product samples during storage. Control fermented dairy product without iodine fortification; KI, sample fortified with potassium iodide; I-Cas, sample fortified with iodocasein.

The dynamics of lactic acid bacteria counts in control and fortified samples of the fermented dairy product during refrigerated storage are presented in Figure 3.

The lower survival rate of viable lactic acid bacteria observed in the KI sample may indicate a reduction in the stability of the fermented milk product matrix in the presence of inorganic iodine. This interpretation is consistent with the previously noted higher post-acidification indices, increased syneresis and reduced viscosity for this variant, which indicate less favourable conditions for maintaining the viability of the starter culture during refrigerated storage.

Maintaining the viability of the starter culture is particularly important for preserving the technological and organoleptic quality of fermented milk products throughout their shelf life. In this regard, the improved survival of lactic acid bacteria observed in the I-Cas sample suggests that the protein-bound form of iodine may provide better compatibility with the milk matrix and, consequently, may be more suitable for the development of fermented dairy products with enhanced stability during refrigerated storage and functional properties.

The results thus suggest that the chemical form of iodine is important not only for micronutrient fortification, but also for maintaining the microbiological stability of fermented dairy products during storage.

### 3.5. Iodine Detection and Quantification

The iodine content in both fortified samples decreased during storage; however, the losses in the KI sample were more pronounced than in the I-Cas variant (Table 2). This indicates that the chemical form of the micronutrient determined not only its effect on the technological properties of the fermented dairy product but also the stability of iodine within the product matrix.

Winger et al. [63] reported that iodine fortification of dairy products is technologically challenging because iodine compounds are chemically reactive and susceptible to losses during processing and storage. Mikláš [64] also noted that iodine concentration in dairy products depends on multiple factors, including production conditions and processing characteristics. In the present study, the differences in iodine retention between the fortified samples indicate that both the dairy matrix and the chemical form of iodine affected micronutrient preservation during storage.

Similar conclusions were reported by Walther et al. [65], who showed that iodine content in milk varies significantly depending on production conditions and season. The authors also highlight differences between organic and conventional milk, confirming the high sensitivity of the iodine profile of dairy products to external and technological factors. Against this background, the higher iodine retention observed in the iodocasein sample can be interpreted as a result of more effective retention of the micronutrient within the protein matrix of the product.

A comparable observation was reported by Niero et al. [66], who emphasized the substantial variability in iodine concentration in milk, ricotta, and yogurt, and noted lower iodine levels in yogurt compared to the raw material. In this context, the higher iodine retention in the I-Cas sample appears technologically justified and may be attributed to the more effective incorporation of the protein-bound form of iodine into the protein matrix, resulting in reduced losses during whey separation. Importantly, this interpretation is supported by the findings of the present study on syneresis: the KI sample, which exhibited more pronounced whey separation, also showed greater iodine losses during storage.

The retention of iodine during storage is a key parameter in the development of fortified dairy products, as losses of micronutrients directly affect the product’s ability to ensure the intended dietary intake of iodine. The higher stability of iodine observed in the I-Cas sample suggests that the protein-bound form of iodine may provide more reliable iodine delivery throughout the storage period compared to potassium iodide.

These findings are particularly relevant for the development of formulations for functional fermented dairy products intended for preventive nutrition programmes aimed at reducing iodine deficiency. Improved iodine retention may contribute to greater consistency between the declared and actual iodine content in the product at the end of its shelf life, which is important for both technological standardisation and nutritional efficacy.

### 3.6. Hedonic Sensory Evaluation

**Figure 4:**
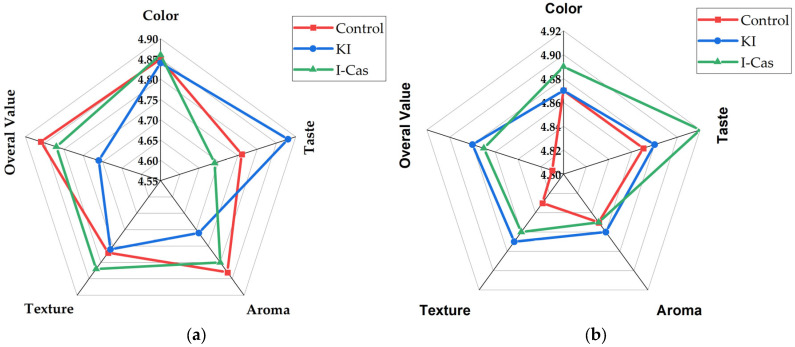
Average value of organoleptic evaluation using hedonic test (**a**) the first day experiment, (**b**) the seventh day experiment. Five-point hedonic scale: 1—dislike very much, 2—dislike moderately, 3—neutral, 4—like moderately, 5—like very much. Control fermented dairy product without iodine fortification; KI, sample fortified with potassium iodide; I-Cas, sample fortified with iodocasein.

The hedonic test is based on a scale with the following categories: “dislike very much,” “dislike,” “neither like nor dislike,” “like,” and “like very much”. This method for assessing product acceptability is among the most widely used. The mean sensory evaluation scores obtained using the hedonic test are presented in Figure 4.

The obtained results indicate a pronounced dependence of consumer properties on sample type and storage duration. On day 1, all samples demonstrated relatively high levels of sensory acceptability (within the range of “like moderately” to “like very much”).

The control sample was characterized by the highest scores for aroma (~4.8) and texture (~4.7), as well as a high overall acceptability (~4.75), indicating good balance and sensory properties familiar to consumers. The scores for color and taste were also high (~4.8 and ~4.7, respectively).

The sample containing KI achieved the highest taste score on day 1 (~4.8), which may be due to the moderate flavour-enhancing effect of potassium iodide at the concentration used and its limited impact on the product’s overall flavour profile. At the same time, the sample with KI showed slightly lower scores for aroma (~4.6) and overall acceptability (~4.6), despite comparable colour scores (~4.8). This may indicate that, although the additive did not have a negative effect on taste perception, it may have affected the volatile aromatic compounds or caused a slight aftertaste that influenced the overall sensory impression.

In contrast, the I-Cas sample demonstrated higher scores for certain attributes. In particular, color (~4.9) and texture (~4.8) were rated higher than in the control, while overall acceptability (~4.75) was at a comparable level. At the same time, taste (~4.6) remained slightly lower, which may be associated with formulation characteristics or ingredient interactions.

Overall, at the initial stage of storage, all samples were well accepted by the panelists; however, the control and I-Cas samples showed a slight advantage in terms of overall performance.

By day 7 of storage, the control sample exhibited the most pronounced decline in sensory scores, particularly for texture (~4.0) and overall acceptability (~4.1). The scores for taste (~4.2) and aroma (~4.3) also decreased, which may be associated with structural retrogradation, moisture loss, and changes in volatile compounds during storage [66].

The KI sample showed relatively stable values compared to the control. Despite a moderate decline, the scores for texture (~4.4) and overall acceptability (~4.3–4.4) remained higher than those of the control sample. This may indicate a potential stabilizing effect of the additive, influencing the structural and water-holding properties of the product [67].

The most stable results were observed in the I-Cas sample. It maintained high scores for color (~4.9), taste (~4.5), and overall acceptability (~4.5), indicating better preservation of sensory characteristics during storage. The slight decrease in aroma and texture (~4.3–4.4) was also less pronounced compared to the control.

The obtained results are consistent with literature data indicating that formulation modification or the incorporation of functional ingredients contributes to improved stability of sensory properties during storage. A number of studies have shown that the addition of bioactive components or structuring agents can slow down texture deterioration and help maintain acceptable taste and aroma throughout storage [68].

The decline in sensory scores observed in the control samples by day 7 is also consistent with general trends reported in the literature, where deterioration in texture and aroma is associated with physicochemical changes in the product matrix. According to Absalimova et al. [69], the incorporation of functional ingredients can affect texture, taste, aroma, and overall acceptability of food products.

The relatively higher stability of the I-Cas sample is in agreement with findings from other authors demonstrating that certain additives can enhance water-holding capacity, stabilize the structure, and slow down the degradation of volatile aroma compounds, thereby maintaining higher consumer acceptability during storage.

The hedonic quality test is used to evaluate key sensory attributes of a product and to obtain information on the level or intensity of these attributes [70].

**Figure 5:**
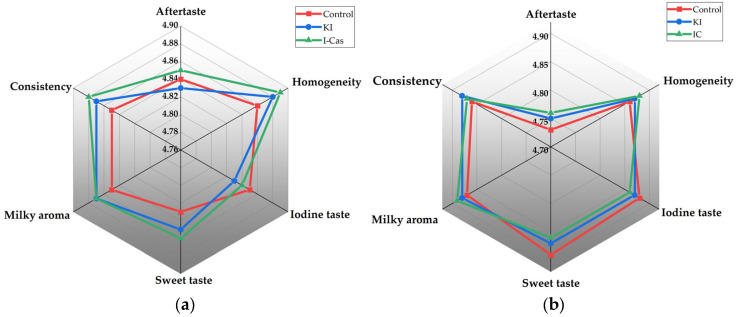
Average value of organoleptic evaluation using hedonic quality test (**a**) the first day experiment, (**b**) the seventh day experiment. Five-point hedonic scale: 1—dislike very much, 2—dislike moderately, 3—neutral, 4—like moderately, 5—like very much. Control fermented dairy product without iodine fortification; KI, sample fortified with potassium iodide; I-Cas, sample fortified with iodocasein. Higher scores for the “iodine taste” attribute correspond to a weaker and more acceptable iodine flavor perception.

The results of the hedonic sensory evaluation demonstrated that all studied samples (Control, KI, and I-Cas) were characterized by relatively high acceptability scores on Day 1 across all assessed attributes, including aftertaste, homogeneity, iodine taste, sweetness, milky aroma, and consistency (Figure 5a,b). For the “iodine taste” attribute, higher scores indicated a more acceptable sensory perception associated with the absence or only a faint, non-offensive iodine flavor. The radar plots indicate that the scores were generally within the upper range of the hedonic scale (≈4.7–4.9), suggesting good initial consumer acceptance.

After 7 days of storage, only minor changes in sensory perception were observed. The overall profiles remained stable, with no drastic decline in acceptability. However, a slight decrease in aftertaste and sweetness scores was noticeable for the Control sample, whereas KI and I-Cas maintained or slightly improved their ratings in homogeneity and iodine taste. Notably, the consistency attribute showed a tendency toward improvement in the experimental samples (KI and I-Cas), which may indicate structural stabilization during storage.

These findings suggest that the developed samples exhibit good sensory stability during storage, with the experimental formulations demonstrating better retention of desirable sensory attributes than the Control.

The observed high initial hedonic scores are consistent with previous research on dairy products, where sensory attributes such as sweetness, aroma, and texture are key drivers of consumer lipping [71]. This aligns with the present results, in which samples with higher sweetness and a milky aroma (KI and I-Cas) demonstrated better hedonic performance.

Regarding storage effects, the relatively minor changes observed between Day 1 and Day 7 contrast with studies on traditional dairy products, where a significant decline in sensory scores during storage has been reported. For instance, in dairy confections, overall acceptability decreased substantially over time due to physicochemical and microbiological changes [72]. In comparison, the stability observed in the current study indicates that the product formulation effectively preserves sensory quality over at least 7 days.

Overall, the hedonic evaluation revealed that the tested samples maintained high sensory acceptability throughout storage, with only slight variations in specific attributes. Compared to literature data, the products demonstrated superior short-term sensory stability, particularly in maintaining aroma, texture, and taste characteristics. The enhanced performance of experimental samples (KI and I-Cas) suggests that the applied formulation or processing approach may contribute to improved sensory quality and storage stability, making them promising candidates for further development and industrial application.

### 3.7. Amino Acid Composition

**Figure 6:**
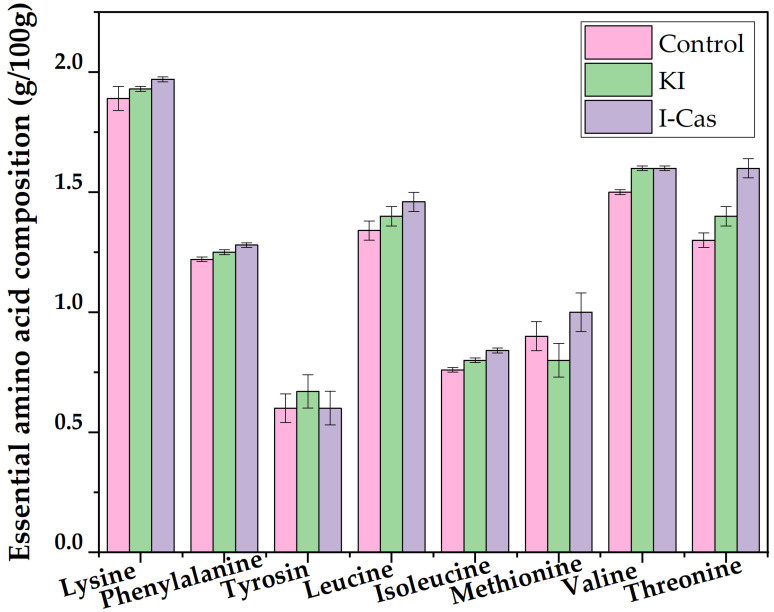
Content of essential amino acids. Control was a fermented dairy product without supplements, KI—potassium iodide, I-Cas—iodocasein.

Analysis of the amino acid composition showed that all samples of fermented dairy products exhibited comparable profiles for both essential (Figure 6) and non-essential amino acids (Table 3). Statistically significant differences between the control samples and the iodine-enriched samples were insignificant (*p* > 0.05).

This result is consistent with the experimental design, as all samples were produced from the same milk raw material, and potassium iodide and iodocasein were added in small quantities, which, as expected, did not affect the overall protein composition of the product. Enrichment with potassium iodide and iodocasein primarily affected the mineral composition of the fermented milk product formulation.

As noted by García-Burgos et al. [73] and Yessenova et al. [74], the impact of fortification on the properties of fermented dairy products largely depends on the nature of the added ingredient: the most pronounced changes in protein, peptide, and structural-functional profiles are typically observed when protein-based, plant-derived, or hydrolyzed ingredients are used, whereas low-dose micronutrient fortification does not necessarily lead to such shifts.

Studies by Abdel-Hamid et al. [75] and Metwalli et al. [76] have shown that the incorporation of protein hydrolysates can modify the peptide and amino acid composition, stimulate the growth of starter microflora, and simultaneously influence the textural characteristics of fermented dairy products. Against this background, the absence of significant differences in the amino acid profile observed in the present study should be considered a methodologically and technologically justified result.

Although no significant differences were found in the amino acid composition of the Control, KI, and I-Cas samples, this does not exclude possible modifications in the structural organization and functional behavior of milk proteins. In particular, iodocasein is a chemically modified form of casein in which iodine is bound predominantly to tyrosine residues [77]. Such modification may influence intermolecular interactions, hydration properties, protein aggregation behavior, and the secondary or tertiary organization of the protein matrix without substantially altering the overall amino acid composition. These structural effects could partly explain the improved viscosity retention and lower syneresis observed in the I-Cas sample during storage. Similar observations have been reported in studies demonstrating that modifications of casein molecules can affect gel formation, water-holding capacity, and rheological properties of fermented dairy systems even when the total amino acid profile remains unchanged [78].

The obtained data indicate that iodine fortification at the applied dosage did not lead to significant alterations in the protein matrix of the product and did not adversely affect its amino acid profile.

## 4. Conclusions

The present study demonstrated that the form of iodine used for fortification influenced the storage behaviour of the fermented dairy product. Differences between potassium iodide and iodocasein became evident primarily during refrigerated storage, particularly in relation to whey separation, viscosity changes, viability of starter microorganisms, and iodine preservation.

Samples fortified with iodocasein showed lower syneresis, better viscosity retention, and higher iodine stability compared with products containing potassium iodide. In addition, the iodocasein formulation maintained microbiological characteristics closer to those of the non-fortified control throughout storage. These observations suggest that the interaction of protein-bound iodine with the dairy matrix may contribute to improved retention of both structural and nutritional properties.

At the fortification level applied in this study, iodine addition did not substantially alter the basic composition or amino acid profile of the fermented dairy product. The obtained results indicate that iodocasein may be a suitable ingredient for the development of fermented dairy products intended for dietary iodine supplementation while maintaining acceptable technological and storage characteristics.

Further research should assess the performance of iodocasein in other fermented dairy matrices and during longer storage periods, including the bioavailability of iodine after consumption.

## Figures and Tables

**Table 1:** Changes in active and titratable acidity of the fermented dairy product during storage (mean ± SD, *n* = 3).

Day of Storage	pH			Titratable Acidity, °T		
	Control	KI	I-Cas	Control	KI	I-Cas
1	4.64 ± 0.02 ^aA^	4.63 ± 0.02 ^aA^	4.65 ± 0.02 ^aA^	78.4 ± 1.4 ^aA^	81.2 ± 1.5 ^aA^	79.3 ± 1.4 ^aA^
3	4.39 ± 0.03 ^aB^	4.33 ± 0.02 ^aB^	4.38 ± 0.03 ^aB^	85.6 ± 1.6 ^bB^	90.1 ± 1.7 ^aB^	86.8 ± 1.5 ^bB^
5	4.33 ± 0.02 ^aB^	4.25 ± 0.03 ^bC^	4.31 ± 0.02 ᵃ^C^	92.7 ± 1.8 ^bC^	99.4 ± 1.9 ᵃ^C^	94.0 ± 1.7 ^bC^
7	4.26 ± 0.03 ᵃ^C^	4.17 ± 0.02 ^bD^	4.24 ± 0.03 ^aD^	100.3 ± 1.9 ^bD^	108.2 ± 2.0 ^aD^	102.1 ± 1.8 ^bD^

Note: Different lowercase letters within the same row and for the same parameter indicate statistically significant differences between samples at *p* < 0.05; Different uppercase letters within the same column and for the same parameter indicate statistically significant differences between storage days at *p* < 0.05. Control fermented dairy product without iodine fortification; KI, sample fortified with potassium iodide; I-Cas, sample fortified with iodocasein.

**Table 2:** Iodine content in the fermented dairy product during storage (µg/100 g, Mean ± SD, *n* = 3).

Days	Control	KI	I-Cas
1	8.2 ± 0.22 ^bA^	24.7 ± 0.41 ^aA^	24.9 ± 0.35 ^aA^
3	7.9 ± 0.20 ^cAB^	23.1 ± 0.37 ^bB^	24.0 ± 0.31 ^aB^
5	7.7 ± 0.21 ^cB^	21.9 ± 0.34 ^bC^	23.5 ± 0.29 ^aB^
7	7.4 ± 0.19 ^cC^	20.5 ± 0.39 ^bD^	23.0 ± 0.33 ^aC^

Note: Control fermented dairy product without iodine fortification; KI, sample fortified with potassium iodide; I-Cas, sample fortified with iodocasein. Different lowercase letters within the same row and for the same parameter indicate statistically significant differences between samples at *p* < 0.05; Different uppercase letters within the same column and for the same parameter indicate statistically significant differences between storage days at *p* < 0.05.

**Table 3:** Amino acid composition of fermented dairy product samples, g/100 g (Mean ± SD).

Treatment	Control	KI	I-Cas
Amino Acids	Non-Essential Amino Acids		
Serine	0.130 ± 0.040	0.140 ± 0.040	0.140 ± 0.040
Glutamic acid	0.430 ± 0.120	0.430 ± 0.120	0.440 ± 0.120
Aspartic acid	0.118 ± 0.040	0.122 ± 0.040	0.126 ± 0.040
Arginine	0.080 ± 0.030	0.080 ± 0.030	0.090 ± 0.040
Histidine	0.060 ± 0.006	0.067 ± 0.007	0.060 ± 0.007
Alanine	0.072 ± 0.030	0.075 ± 0.030	0.078 ± 0.030
Glycine	0.041 ± 0.020	0.043 ± 0.020	0.045 ± 0.020

Note: Control fermented dairy product without iodine fortification; KI, sample fortified with potassium iodide; I-Cas, sample fortified with iodocasein.

## Data Availability

The original contributions presented in the study are included in the article. Further inquiries can be directed to the corresponding authors.

## Abbreviations

The following abbreviations are used in this manuscript:

Control	control sample
KI	fermented dairy product fortified with potassium iodide
I-Cas	fermented dairy product fortified with iodocasein
ANOVA	analysis of variance
SD	standard deviation
CFU	colony-forming unit
ICP-MS	inductively coupled plasma mass spectrometry
